# Development and validation of a glioma-associated mesenchymal stem cell-related gene prognostic index for predicting prognosis and guiding individualized therapy in glioma

**DOI:** 10.1186/s13287-023-03285-9

**Published:** 2023-04-01

**Authors:** Zesheng Peng, Yuxi Wu, Jiajing Wang, Sujie Gu, Yihao Wang, Bingzhou Xue, Peng Fu, Wei Xiang

**Affiliations:** Department of Neurosurgery, Union Hospital, Tongji Medical College, Huazhong University of Science and Technology, Wuhan, Hubei China

**Keywords:** Glioma-associated mesenchymal stem cells, Glioma, Prognosis, Chemoradiotherapy, Immunotherapy, Immune microenvironment, Genomic alterations

## Abstract

**Background:**

Recent studies have demonstrated that glioma-associated mesenchymal stem cells (GA-MSCs) are implicated in the regulation of glioma malignant progression. However, the prognostic value of GA-MSCs has not been comprehensively explored in glioma.

**Methods:**

We extracted GA-MSCs from glioma tissues, established intracranial xenograft models in nude mice, and obtained GA-MSC-related genes (GA-MSCRGs) by using microarrays. The transcriptome data and clinical information of glioma patients were obtained from the CGGA and TCGA databases. We screened 8 prognostic GA-MSCRGs to construct a prognostic index by using the multivariate Cox regression method. The validity of the GA-MSCRGPI was verified in the training (CGGA693) and validation (TCGA and CGGA325) cohorts. The expression patterns of these 8 GA-MSCRGs were validated in 78 glioma tissue specimens by using a qRT‒PCR assay.

**Results:**

GA-MSCs were successfully isolated from glioma tissues. Based on intracranial xenograft models and transcriptome microarray screening, 8 genes (MCM7, CDK6, ORC1, CCL20, TNFRSF12A, POLA1, TRAF1 and TIAM1) were selected for the construction of a GA-MSC-related gene prognostic index (GA-MSCRGPI). In both the training and validation cohorts, high GA-MSCRGPI patients showed an inferior survival outcome compared with low GA-MSCRGPI patients. A nomogram was established based on independent prognostic indicators (age, WHO grade and GA-MSCRGPI) and exhibited a strong forecasting ability for overall survival (OS). Moreover, we found that the GA-MSCRGPI could evaluate the prognosis of glioma patients undergoing chemoradiotherapy. The high GA-MSCRGPI group exhibited higher immune, stromal and ESTIMATE scores; lower tumor purity; higher infiltration of Tregs and M2-type macrophages; fewer activated NK cells; and higher expression of immune checkpoints. Tumor Immune Dysfunction and Exclusion (TIDE) showed that the high GA-MSCRGPI group had more responders to ICI therapy. The results of the genetic mutation profile and tumor mutation burden (TMB) in different GA-MSCRGPI subgroups further supplement GA-MSCRGPI-related mechanisms. Finally, the expression patterns of 8 selected GA-MSCRGs in GA-MSCRGPI were correlated with glioma WHO grades to a certain extent.

**Conclusion:**

The constructed GA-MSCRGPI could predict prognosis and guide individualized therapy in glioma patients.

**Supplementary Information:**

The online version contains supplementary material available at 10.1186/s13287-023-03285-9.

## Background

Malignant gliomas are the most frequent primary tumor with a high recurrence rate and high mortality in the central nervous system [1]. Because of the highly intratumoral heterogeneity that leads to diverse invasiveness, aggressiveness and treatment responsiveness [2], the accurate diagnosis of glioma for predicting therapeutic response and prognosis remains a major clinical challenge. Recent advances in the molecular pathology of gliomas have brought a glimmer of hope [3]. Molecular biomarkers, represented by isocitrate dehydrogenase (IDH) mutation [4], 1p19q codeletion [5] and MGMT promoter (MGMTp) methylation [6], were updated to guidelines and partly benefited patients; however, nonnegligible heterogeneities still exist even in the same subtype. As a critical factor in determining heterogeneity, the tumor microenvironment (TME) further contributes to the malignant biological behavior of glioma in a cancer cell-extrinsic manner [7, 8]. In addition to traditional components, including astrocytes, endothelia, cancer-associated fibroblasts (CAFs) and immune cells [9], recent evidence indicates that glioma-associated mesenchymal stem cells (GA-MSCs) may also participate in the formation of the TME [10].

GA-MSCs are a kind of stromal cell subset isolated from glioma tissue that show plastic and adherent morphology in vitro without tumorigenicity in vivo. It expresses CD105, CD90, CD73, CD44 and other mesenchymal stem cell markers, and it also differentiates into adipocytes, osteoblasts and chondrocytes under specific stimulation [11]. Previous studies have found that GA-MSCs promote proliferation and maintain the stemness of glioma stem cells (GSCs) through the IL-6/gp130/STAT3 pathway [10]. It also increases the tumorigenicity of GSCs by secreting miR-1587-containing exosomes [12]. Furthermore, based on the expression of CD90 on the cytomembrane [13], our team divided GA-MSCs into two subpopulations, in which the CD90^high^ group increased the proliferation, migration and invasion of glioma cells, while the CD90^low^ group not only regulated angiogenesis via pericyte transition but also promoted temozolomide resistance by activating FOXS1-mediated epithelial-mesenchymal transition [14, 15]. In general, GA-MSCs have been shown to positively potentiate glioma progression and may be an independent prognostic factor for poorer outcomes in glioma.

A previous study reported that the percentage of cells coexpressing CD105 + /CD73 + /CD90 + in glioma tumor samples inversely correlates with patient overall survival [16]; however, some limitations and deficiencies should be addressed. First, although CD105, CD73 and CD90 are representative molecular markers of MSCs [17], their specificity is not strong enough. Since these markers are frequently expressed in endothelial cells [18–20] and negative markers of MSCs were not considered at the same time, the method of this study remains questionable. Second, this study only explored the association between the percentage of MSCs and patient overall survival (OS) and ignored other important prognostic indicators; therefore, it lacks sufficient clinical guiding significance and application value. Last but not least, although GA-MSCs account for a high proportion of high-grade gliomas, there are also a certain number of GA-MSCs in low-grade gliomas [10, 14]. Compared with the overall poor prognosis of high-grade glioma patients, the prognosis of low-grade glioma is more significantly different [21]. Thus, it is equally important to explore the relationship between GA-MSCs and the prognosis of low-grade glioma patients.

In this study, we extracted GA-MSCs from glioma tissues, established intracranial xenograft models in nude mice, and obtained differential genes, namely GA-MSC-related genes (GA-MSCRGs), through transcriptome microarrays combined with Gene Set Enrichment Analysis (GSEA) enrichment analysis. Then, we screened 8 prognostic GA-MSCRGs to construct a prognostic index (GA-MSCRGPI) and systematically evaluated its prognostic value and predictive value in the efficacy of chemoradiotherapy. Moreover, its correlations with the immune landscape and possibility of application in immune checkpoint inhibition (ICI) therapy were also investigated. Finally, the expression patterns of these 8 GA-MSCRGs were validated in 78 glioma tissue specimens using a qRT‒PCR assay. We aimed to comprehensively assess the correlation of GA-MSCs with prognosis, the tumor microenvironment, and therapeutic efficacy in gliomas.

## Methods

### Isolation and culture of GA-MSCs

This study was approved by the Institutional ethics committee of Tongji Medical College, Huazhong University of Science and Technology. As we described previously, GA-MSCs were obtained from tumor specimens of glioma patients who provided informed consent [14]. The clinical indicators of these patients were shown in Additional file 1: Table S1 (Inpatient No. 3100630, 3058653 and 3044554). In brief, fresh glioma specimens were washed with phosphate-buffered saline (PBS, HyClone, USA) and then processed for mechanical cutting and trypsin (Biosharp, China) digestion. After filtering with a 70-µm nylon mesh and removing erythrocytes, the mononuclear cells were collected by Ficoll (2:1 Genview, USA) density gradient centrifugation and cultured in DMEM (HyClone, USA) containing 10% fetal bovine serum (FBS; BI, Israel), 2 mM L-glutamine (Beyotime, China), and 100U/ml penicillin and 0.1 mg/ml streptomycin (Biosharp, China) in a humidified atmosphere at 37 °C containing 5% CO2. After 24 h, nonadherent cells were removed, and adherent cells were cultured until they reached confluence. GA-MSCs were passaged using Accutase (Gibco, USA) and used for subsequent experiments at passages 2 to 3.

### Identification of GA-MSCs

For the identification of GA-MSCs, flow cytometry and induced differentiation were performed as we described previously [14]. In brief, GA-MSCs were collected and stained with fluorochrome-conjugated antibodies, including anti-CD105-APC, anti-CD90-PerCP, anti-CD73-APC/Cy7, anti-CD44-FITC, anti-CD133-APC and anti-CD34-APC (all from eBioscience. USA) in the dark at 4 °C for 30 min. Then, the cells were centrifuged, resuspended and analyzed using a FACS flow cytometer (BD FACSCanto2, Biosciences). The data were collected and analyzed using FlowJo software (v10.6.2). GA-MSCs were differentiated into osteocytes, adipocytes, and chondrocytes by using ready-to-use differentiation media (all from Cyagen, China) following the manufacturer’s instructions. Adipogenic differentiation was evaluated by Oil Red O staining, osteogenic differentiation was evaluated by Alizarin Red staining, and chondrogenic differentiation was evaluated by Alcian Blue staining. The stain results were observed with an inverted phase contrast microscope (Olympus IX73), and photographs were taken with a digital camera using Image-Pro Plus 6.0 software.

### Intracranial xenograft model

A total of 25 6-week-old male Balb/C nude mices were purchased from Shulaibao Biotechnology Co., Ltd. (Wuhan, China). Animals were divided into three groups by simple randomization and reared under the same conditions for 3 days. To construct an intracranial xenograft model, conventional U87-MG cells and identified GA-MSCs were resuspended and mixed at a ratio of 5:1 and then injected (5 µl cell suspension, a total of 5X10^5^ cells) into the right frontal lobe of nude mices (n = 10) using a Hamilton syringe (Hamilton Company, USA). U87-MG cells or GA-MSCs implanted alone served as negative controls (n of U87-MG group = 10, n of GA-MSCs group = 5). Animals that developed intracranial tumors after injection and survived for a certain amount of time were included in the statistics, otherwise were excluded. For the survival analysis, the mice were monitored periodically and sacrificed when they showed severe neurological symptoms or obvious cancerous cachexia. The whole brains of the mice were removed, and some samples were fixed in 4% paraformaldehyde and then embedded in paraffin for pathologic analysis. The other isolated tumor samples were kept into MACS Tissue Storage Solution (Miltenyi, Germany) for subsequent experiments. Mices were anesthetized by intraperitoneal injection of sodium pentobarbital (35 mg/kg) and final euthanized by cervical dislocation under deep anesthesia. All animal procedures were conducted in accordance with institutional guidelines under approved protocols.

### RNA extraction and Clariom D microarray

Total RNA from isolated tumors was extracted using TRIzol reagent (Invitrogen, USA) according to the manufacturer's protocol. After the total RNA quality was analyzed, cDNA was prepared using the GeneChip WT PLUS Kit and hybridized onto GeneChip® Clariom D arrays (Affymetrix, USA). After washing and scanning according to the manufacturer's instructions, the microarray data were measured and summarized using Clariom D QC tool software (Affymetrix, USA).

### Data screening and collection from a public database

RNA sequencing (RNA-seq) data and clinical information of glioma patients were extracted from the Chinese Glioma Genome Atlas (CGGA; http://www.cgga.org.cn/) and The Cancer Genome Atlas (TCGA; https://portal.gdc. cancer.gov/) databases. Cases without survival data or overall survival < 30 days or without definitive histopathological diagnosis were excluded. Eventually,

A total of 1557 patients were enrolled in subsequent studies, of which 655 patients from the CGGA database (mRNAseq_693 dataset) served as the training cohort, and 305 patients from the CGGA database (mRNAseq_325 dataset) and 597 patients from the TCGA database were defined as the validation cohorts. All RNA-seq data were obtained in the format of fragments per kilobase of exon model per million mapped reads (FPKM) normalized. The clinicopathological characteristics of all included patients were summarized in Additional file 1: Table S2.

### Identification of prognostic GA-MSC-related genes

Following the microarray data, differentially expressed genes (log_2_|fold change (FC)|> 1 and adjusted *P* values < 0.05) were selected from the mixed implanted group and U87-MG control group. Next, GSEA was carried out to uncover the functional association of these genes and further screen out key roles. In addition, univariate Cox regression analysis was subsequently performed for prognostic identification (*P* < 0.05), and prognostic genes shared by the three cohorts were considered eligible. Eventually, 45 differentially expressed genes were obtained as prognostic GA-MSCRGs.

### Construction and validation of the GA-MSCRGPI

The prognostic GA-MSCRGs in the training cohort were incorporated into the least absolute shrinkage and selection operator (LASSO) Cox regression by using the R package “glmnet”. The minimum tenfold cross-validation was used to select the optimal value of λ, and 18 GA-MSCRGs were identified. Subsequently, multivariate Cox regression was performed to construct the GA-MSCRGPI. The calculation formula of GA-MSCRGPI is shown as follows: :

$${\text{GA-MSCRGPI}} = \sum\limits_{i = 1}^{{{n}}} {Coef_{{i }} } *\chi_{ i }$$

where* χ*_*i*_ and *Coef*_*i*_ refer to the expression level of selected GA-MSCRGs and the corresponding coefficient in the Cox model, respectively. The median GA-MSCRGPI was used as the cutoff value for stratifying patients into the high/low GA-MSCRGPI subgroup. The Kaplan‒Meier curve with the log-rank test was plotted by using the R package survminer for the comparison of OS between GA-MSCRGPI subgroups. Receiver operating characteristic (ROC) curve analysis was utilized to quantify the prognostic power of GA-MSCRGPI via the R package “timeROC.” All validation tests were performed in both the training and validation cohorts.

### Establishment and evaluation of a nomogram

Univariate and multivariate Cox regression analyses were used to determine the independent prognostic value of the GA-MSCRGPI. Subsequently, a nomogram based on independent prognostic factors in the training cohort was established by using the R package “rms”. Calibration curves at 2, 3, and 5 years were plotted for graphical evaluation. The concordance index (C-index) and the ROC curve were used to assess the predictive accuracy of the nomogram.

### Evaluation of immune characteristics and ICI therapy response

The immune scores, stromal scores, ESTIMATE scores and tumor purity were measured using the ESTIMATE algorithm via the R package “estimate”. The abundance of 22 immune cells was calculated through the CIBERSORT algorithm with 1000 permutations. In addition, we applied Pearson correlation analysis to calculate the correlation between GA-MSCRGPI and the expression levels of seven immune cell markers and evaluated the differential expression of those markers in subgroups.

### Tissue samples and quantitative real-time polymerase chain reaction (qRT‒PCR)

All human glioma tissues and corresponding clinical information were obtained from the Department of Neurosurgery of Wuhan Union Hospital from July 2017 to July 2021. The clinicopathological characteristics of the patients were summarized in Additional file 1: Table S1 Fresh tumor tissues were resected and immediately preserved in liquid nitrogen. Total RNA was extracted from each sample. According to the manufacturer's instructions, cDNA was synthesized by reverse transcription using a reverse transcription kit (Takara RR036A). qRT‒PCR analysis was further performed on a LightCycler 480 Real-Time PCR system using TB Green® Premix Ex Taq™ II (Takara RR820A). GAPDH was used for normalization, and the comparative Ct method (ΔΔCt) was used to evaluate mRNA expression. The primer sequences are listed in Additional file 3: Table S3.

### Statistical analysis

The downloaded data were organized using Excel software. Data analysis and visualization were performed mainly by R software (v3.6.1). Differences in and clinical characteristics or animal survival were analyzed and visualized by using GraphPad Prism (v9.0.0). Log-rank test was used for survival analysis. The chi-square test was executed for the comparison of categorical variables between GA-MSCRGPI subgroups. Student’s *t* test or one-way ANOVA (followed by Bonferroni post hoc tests) was utilized to compare the continuous variables between two groups or more than two groups. The nonparametric test was used to compare the expression levels of selected GA-MSCRGPIs between glioma tissues. All statistical tests were bilateral, and a *P* value < 0.05 was considered statistically significant.

## Result

### Identification of prognostic GA-MSCRGs

**Fig. 1:**
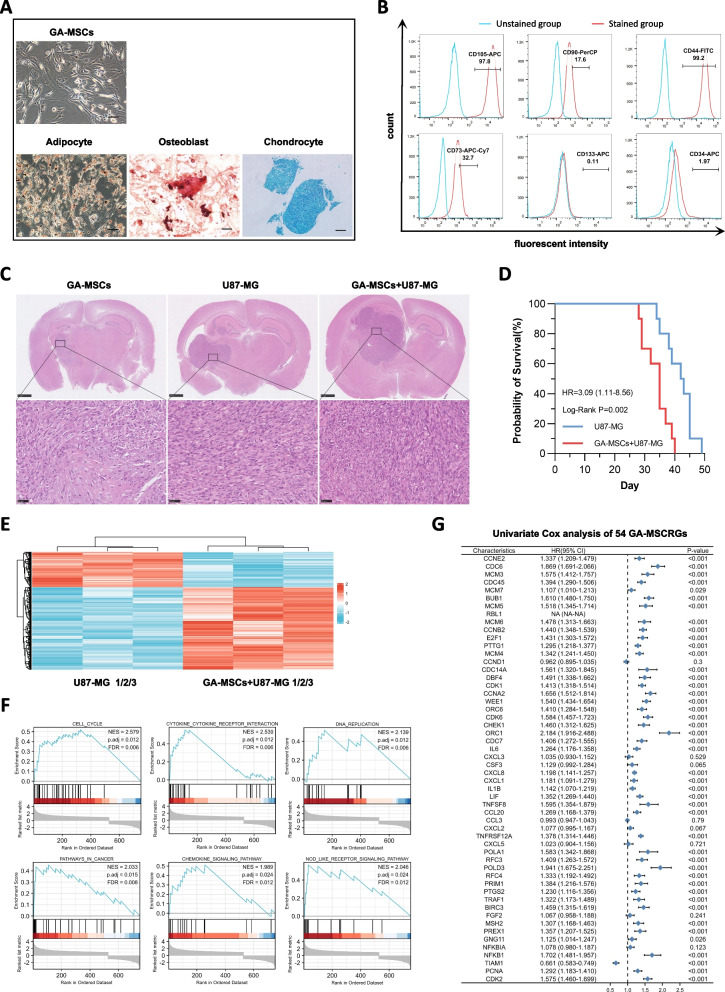
Identification of Prognostic GA-MSCRGs. **A** Morphological characteristics of GA-MSCs cultured in 10% FBS-containing DMEM (× 200, scale bars = 100 µm) and differentiation of GA-MSCs into adipocytes (× 200, scale bars = 100 µm), osteoblasts (× 400, scale bars = 50 µm) and chondrocytes (× 200, scale bars = 100 µm). **B** FACS analysis of typical GA-MSCs in vitro. **C** Construction of intracranial xenograft models with U87-MG cells and GA-MSCs (H&E staining, upper panels: × 25, scale bars = 1 mm; lower panels: × 400, scale bars = 50 µm). **D** Survival curves of intracranial xenograft mice. **E** Heatmap of 814 DEGs from xenograft tumors performed on U87-MG and U87-MG + GA-MSCs cells. “Red” indicates high relative expression, and “Blue” indicates low relative expression. **F** GSEA was performed in 814 DEGs (*p* < 0.05, FDR < 0.25). **G** Univariate Cox regression analysis of 54 DEGs in the CGGA693 cohort (HR, hazard ratio; CI: confidence interval)

In this study, GA-MSCs were successfully isolated from fresh astrocytoma or glioblastoma tissues. These cells exhibited similar classical MSC characteristics in a series of identification experiments. In vitro, GA-MSCs grew adherent to flasks with spindle shape morphology and were able to differentiate into adipocytes, osteoblasts and chondrocytes by using specific stimuli to promote adipogenesis, osteogenesis and chondrogenesis, respectively (Fig. 1A). Moreover, GA-MSCs positively expressed CD105, CD90, CD73 and CD44 but negatively expressed CD133 or CD34 (Fig. 1B). In vivo, GA-MSCs were not tumorigenic, and intracranial implantation of GA-MSCs caused only mild glial hyperplasia. However, when we mixed the glioma cells U87-MG with GA-MSCs at the given ratio and implanted mixtures into the brains of mice, they eventually formed larger tumors relative to U87-MG cells implanted alone (Fig. 1C). The survival time of mice that were implanted with U87-MG cells and GA-MSCs was significantly shorter than that of mice implanted with U87-MG cells alone (Fig. 1D), indicating that GA-MSCs increased the tumorigenesis of glioma cells in vivo. Next, we randomly selected 3 cases from the xenograft mixture as the experimental group and 3 cases from U87-MG xenografts as the control group. Total RNA was extracted and detected using a gene microarray. The results identified a total of 814 differentially expressed genes (DEGs). Among them, 570 DEGs were upregulated and 244 were downregulated in the experimental group (Fig. 1E). Then, GSEA using the predefined Kyoto Encyclopedia of Genes and Genomes (KEGG) set was performed to uncover the functional association of these DEGs and further screen the central roles. Six tumor-associated gene sets, including the cell cycle, cytokine‒cytokine receptor interaction, DNA replication, pathway in cancer, chemokine signaling pathway and NOD-like receptor signaling pathway, were enriched (Fig. 1 F), and 54 DEGs in core enrichment were screened out. In addition, we assessed the prognostic significance of these 54 DEGs by performing univariate Cox regression analysis in both the CGGA and TCGA cohorts (Fig. 1G and Additional file 5: Figure S1). Eventually, a total of 45 OS-associated DEGs were identified as prognostic GA-MSCRGs.

### Construction and validation of the GA-MSCRGPI

**Fig. 2:**
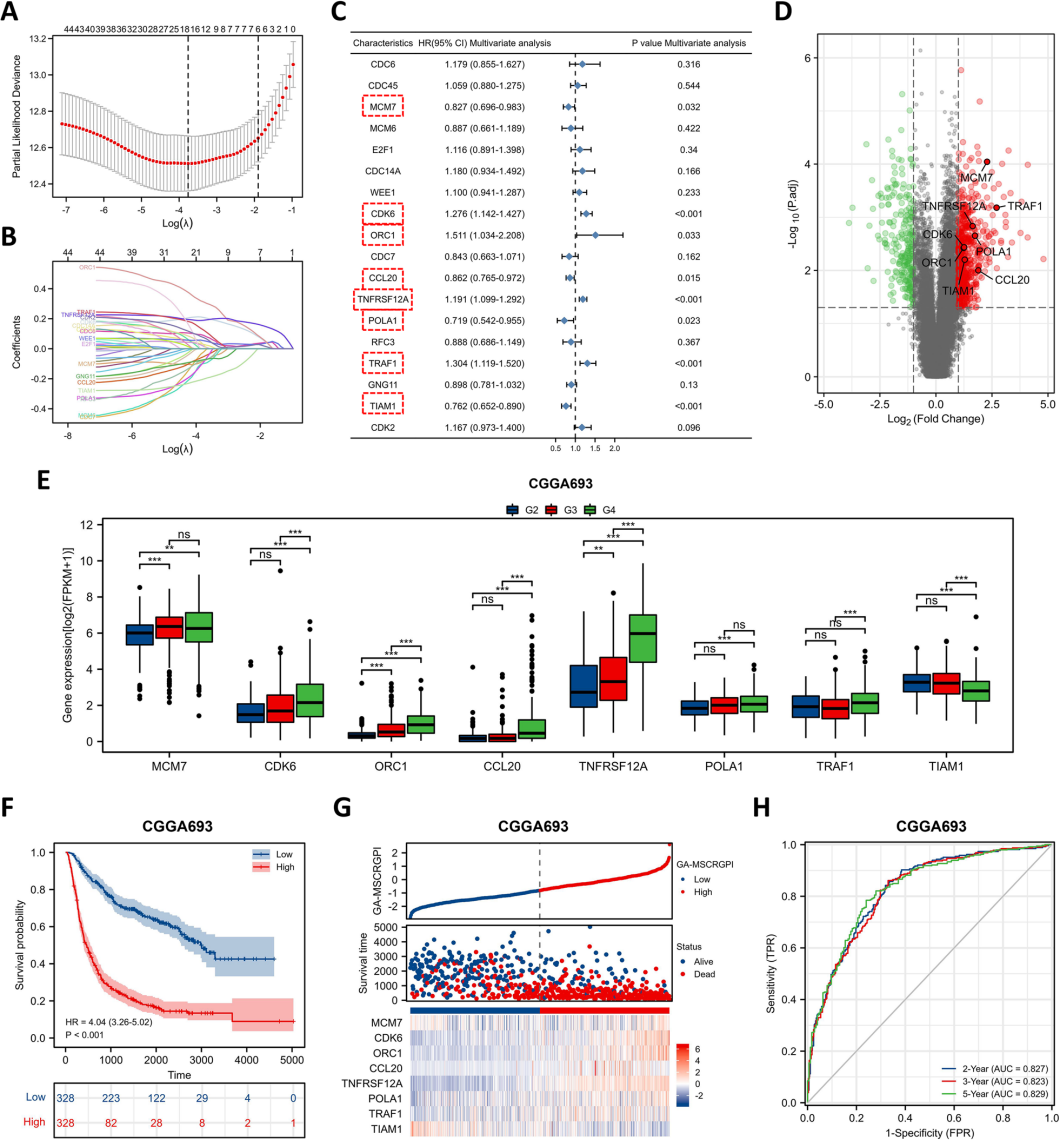
Construction of the GA-MSCRGPI in the CGGA693 cohort. **A, B** LASSO regression was performed with the minimum criteria. **C** Multivariate Cox regression was used to construct a GA-MSCRGPI (8 GA-MSCRGs for modeling are shown in red squares). **D** The volcano map shows the distribution of 8 selected GA-MSCRGs in 814 DEGs (“red” indicates high relative expression, and “green” indicates low relative expression). **E** Expression comparison of 8 selected GA-MSCRGs between different grades of glioma tissues in the CGGA693 cohort (G2: WHO grade II, G3: WHO grade III, G4: WHO grade IV; **p < 0.01, ***p < 0.001, and **ns:** no significance). **F** Kaplan‒Meier curves of GA-MSCRGPI subgroups for survival. **G** The distribution plots of GA-MSCRGPI, survival status and expression of 8 selected GA-MSCRGs. **H** ROC curve analysis of GA-MSCRGPI in predicting 2-, 3- and 5-year OS

These 45 prognostic GA-MSCRGs were incorporated into the least absolute shrinkage and selection operator (LASSO) regression in the CGGA693 cohort (Fig. 2A, B), and eighteen of these were selected for further multivariate Cox regression analysis. Consequently, only eight GA-MSCRGs (MCM7, CDK6, ORC1, CCL20, TNFRSF12A, POLA1, TRAF1 and TIAM1) were independent predictors for OS (Fig. 2C, D). The expression levels of these GA-MSCRGs were correlated with WHO grades to a certain extent in both training (Fig. 2E) and validation (Additional file 6: Figure S2A, E) cohorts. Then, a prognostic index was constructed and calculated by the following formula: GA-MSCRGPI = (−0.260 * expression level of MCM7) + (0.285 * expression level of CDK6) + (0.709 * expression level of ORC1) + (−0.153 * expression level of CCL20) + (0.202 * expression level of TNFRSF12A) + (− 0.293 * expression level of POLA1) + (0.271 * expression level of TRAF1) + (− 0.310 * expression level of TIAM1). Then, patients were categorized into different subgroups using the median GA-MSCRGPI as the cutoff value. In the CGGA693 cohort, the Kaplan‒Meier curve suggested that patients in the low GA-MSCRGPI group had a significantly longer OS than patients in the high GA-MSCRGPI group (Fig. 2F). The GA-MSCRGPI and survival status distributions showed that patients with higher GA-MSCRGPI had shorter OS and more dead status (Fig. 2G). The ROC curves showed that the GA-MSCRGPI had a satisfactory prediction performance (2-year AUC = 0.827, 3-year AUC = 0.823, 5-year AUC = 0.829; Fig. 2H). To further verify the prognostic power of the GA-MSCRGPI, the same analyses were carried out in the TCGA and CGGA325 cohorts. Consistent with the training cohort, patients with a low GA-MSCRGPI had better survival outcomes than patients with a high GA-MSCRGPI in the validation cohorts (Additional file 6: Figure S2B, C, F and G). The ROC curves confirmed the potent capability of GA-MSCRGPI to predict OS in both TCGA cohorts (2-year AUC = 0.868, 3-year AUC = 0.870, 5-year AUC = 0.838; Additional file 6: Figure S2D) and CGGA325 cohort (2-year AUC = 0.849, 3-year AUC = 0.878, 5-year AUC = 0.889; Additional file 6: Figure S2H). All results agreed that the prognostic GA-MSCRGPI could accurately and stably predict the survival outcome of glioma patients.

### Stratification analysis of the prognostic GA-MSCRGPI based on clinical characteristics

**Fig. 3:**
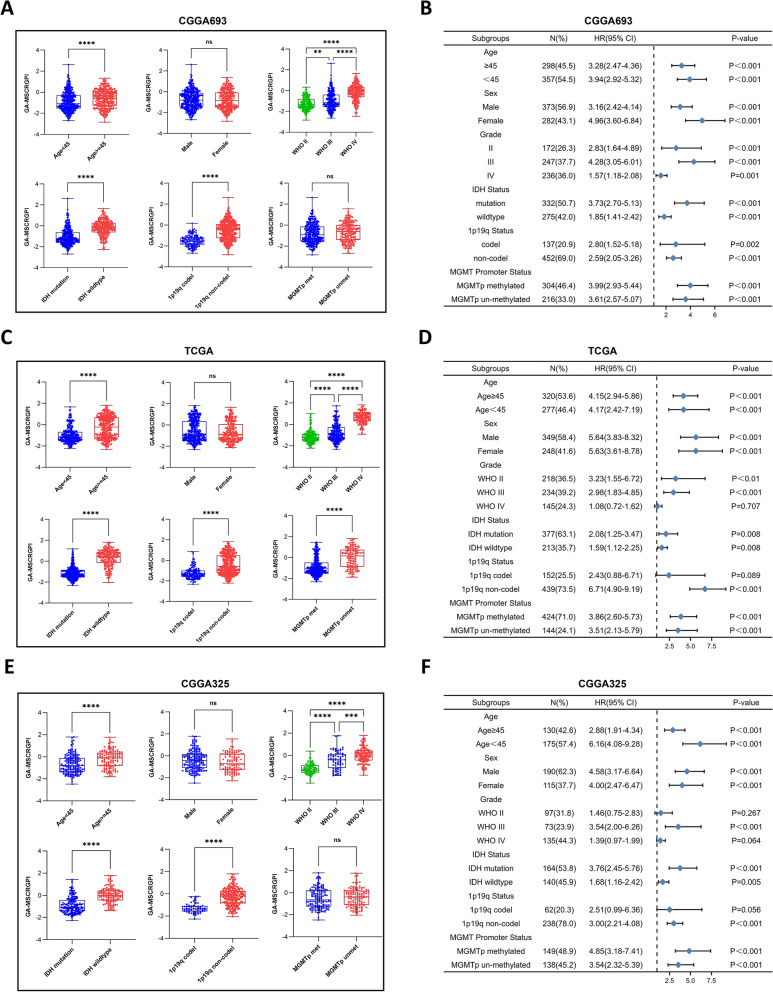
Correlation analysis between the GA-MSCRGPI and clinicopathological characteristics in both the training and validation cohorts. **A, C, E** Different levels of GA-MSCRGPI in glioma patients stratified by age, sex, grade, IDH status, 1p19q codeletion and MGMT promoter status (**p < 0.01, ***p < 0.001, ****p < 0.0001, and ns No significance). **B, D F** Forest maps showing the survival outcomes of subgroups stratified by these clinicopathological characteristics

First, we compared the levels of GA-MSCRGPI between patients stratified by various clinical characteristics, including age, sex, grade, IDH status, 1p19q codeletion, MGMT promoter status and 2016 WHO classification. In the training cohort, patients with the clinical features of age ≥ 45 years, higher grade, IDH wild-type, and 1p19q noncodeletion showed significantly higher levels of GA-MSCRGPI, while no differences were observed between patients stratified by sex and MGMT promoter status (Fig. 3A). The same results were obtained in the validation cohort of CGGA325 (Fig. 3C), while only slight differences were observed in the validation cohort of TCGA. Glioma patients with unmethylated MGMT promoters showed relatively higher levels of GA-MSCRGPI in the TCGA cohort, whereas other results were consistent with those in both CGGA cohorts (Fig. 3E). Furthermore, to determine whether these clinical characteristics would impact the prediction accuracy of the prognostic GA-MSCRGPI, we performed subgroup survival analyses, and the results were presented by using forest maps. In the training cohort, patients with a high GA-MSCRGPI had worse survival outcomes than those with a low GA-MSCRGPI in all subgroups (Fig. 3B). In the validation cohorts, the results of subgroup survival analyses were largely consistent with those in the training cohort, except for the WHO II, WHO IV and 1p19q codel subgroups in the TCGA and CGGA325 cohorts (Fig. 3D, F).

### Construction and evaluation of the nomogram

**Fig. 4:**
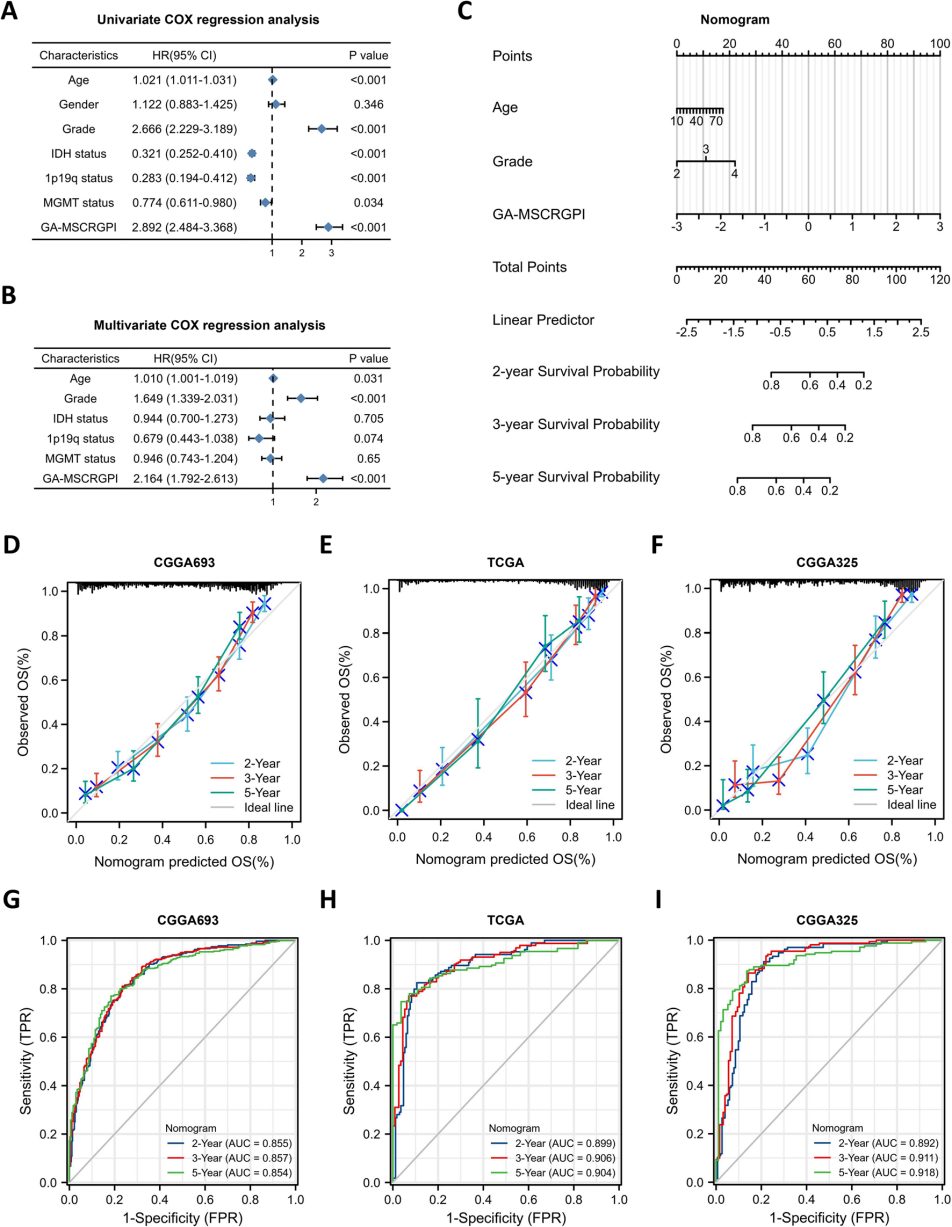
Establishment and evaluation of a nomogram. **A, B** Univariate and multivariate Cox regression analyses in the CGGA693 cohort. **C** Nomogram based on GA-MSCRGPI, age and WHO grade. **D, E, F** Calibration curves showed the concordance between predicted and observed 2-, 3-, and 5-year OS in CGGA693, TCGA and CGGA325. **G, H, I**) ROC curve analyses of the nomogram in predicting 2-, 3-, and 5-year OS in CGGA693, TCGA, and CGGA325

To identify whether the established GA-MSCRGPI can be qualified as a prognostic predictor of glioma, we conducted univariate and multivariate Cox regression analyses in combination with common clinicopathological characteristics. Both in the training and validation cohorts, the GA-MSCRGPI revealed satisfactory prognostic efficiency, such as age, tumor grade, IDH status, 1p19q codel status and MGMT promoter unmethylated status (Fig. 4A). Moreover, the GA-MSCRGPI was also an independent predictor in the multivariate Cox regression analysis (Fig. 4B). These results illustrated that the GA-MSCRGPI could serve as a reliable and novel prognostic biomarker. To make our prognostic GA-MSCRGPI more applicable for clinical use, we subsequently established a nomogram using these independent prognostic factors (age, grade, and GA-MSCRGPI) in the training cohort (Fig. 4C). The internal evaluation was performed by using the concordance index (C-index) and calibration plots, and the external evaluation was conducted by using the same method in the validation cohorts. The C-index of this nomogram was 0.771 in the CGGA693 cohort, 0.846 in the TCGA cohort and 0.780 in the CGGA325 cohort. The calibration plots revealed an excellent match between the actual and nomogram-predicted probability of 2-, 3-, and 5-year OS in both the training and validation cohorts (Fig. 4D, E and F). The ROC curves presented excellent sensitivity and specificity of the prognostic GA-MSCRGPI in both the CGGA693 cohort (2-year AUC = 0.855, 3-year AUC = 0.857, 5-year AUC = 0.854; Fig. 4 G) and TCGA cohort (2-year AUC = 0.899, 3-year AUC = 0.906, 5-year AUC = 0.904; Fig. 4H) and CGGA325 cohort (2-year AUC = 0.892, 3-year AUC = 0.911, 5-year AUC = 0.918; Fig. 4I). These results together confirmed that the nomogram had satisfactory prognostic efficiency for glioma, and it had the potential to be developed into a quantitative tool to predict the prognosis of glioma patients.

### Association between GA-MSCRGPI and efficacy of chemoradiotherapy

**Fig. 5:**
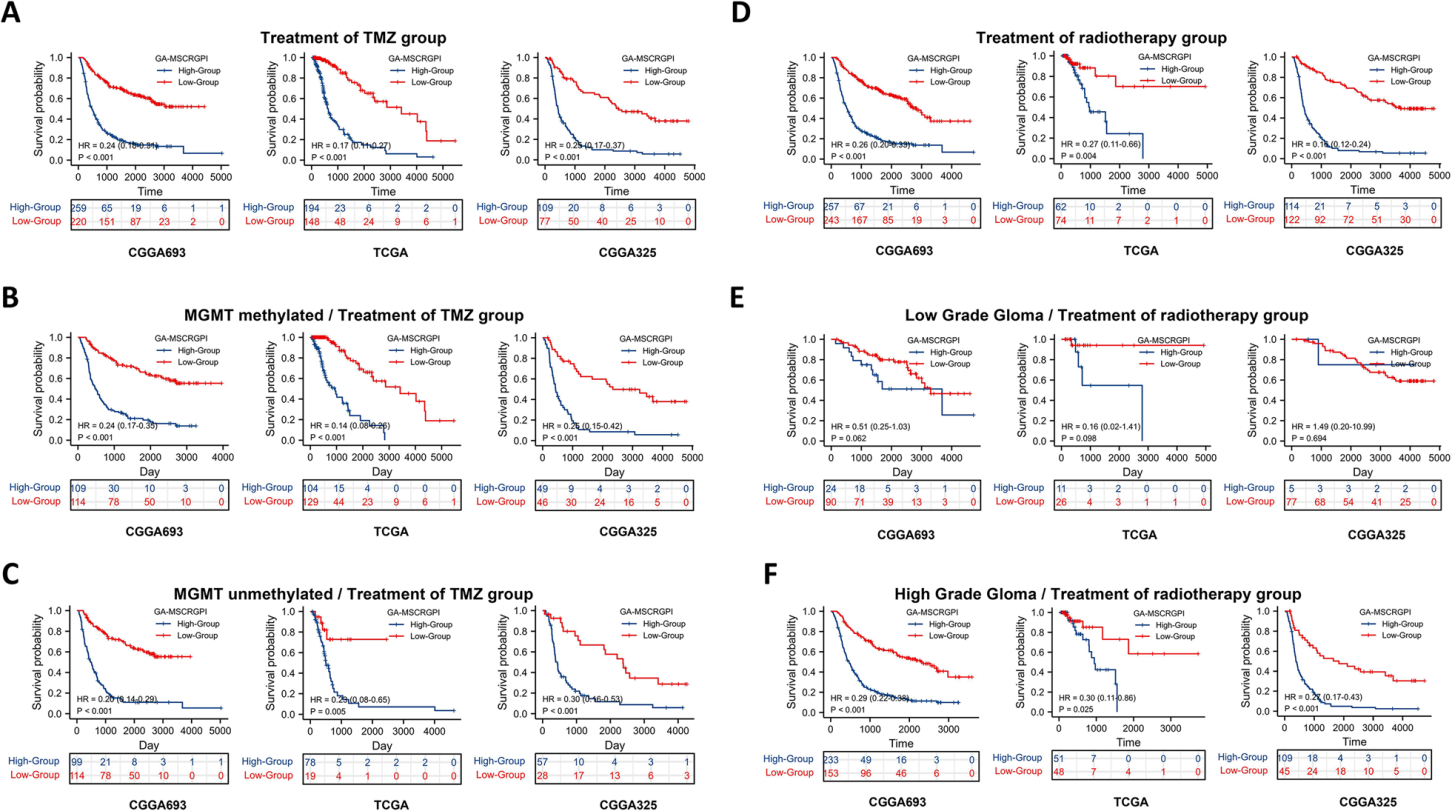
Exploration of the association between GA-MSCRGPI and chemoradiotherapeutic efficacy. **A** Survival outcomes between high and low GA-MSCRGPI subgroups in patients who were treated with TMZ at any time. **B, C** Stratification analysis according to MGMT promoter status. Kaplan‒Meier curves showed survival differences between the high and low GA-MSCRGPI subgroups. **D** The OS between high and low GA-MSCRGPI subgroups in patients with radiotherapy. **E, F** Kaplan‒Meier curves of different GA-MSCRGPI subgroups in low- and high-grade patients with radiotherapy

Previously, we reported that GA-MSCs could promote temozolomide resistance in glioma cells through the epithelial-mesenchymal transition pathway [15]. This result suggested that our established GA-MSCRGPI might be associated with the therapeutic efficacy of glioma. First, we explored the correlation between GA-MSCRGPI-based stratification and the efficacy of chemoradiotherapy (Additional file 7: Figure S3). In the training cohort, the GA-MSCRGPI-based stratification was not correlated with the efficacy of temozolomide chemotherapy or radiotherapy, whereas the results presented relative instability in the validation cohorts. Although no significant survival benefit of chemotherapy or radiotherapy was observed in the TCGA cohort, both chemotherapy and radiotherapy can benefit patients with high GA-MSCRGPI in the CGGA325 cohort. Subsequently, we explored the survival differences of patients who underwent chemoradiotherapy between the high and low GA-MSCRGPI subgroups. The results showed that patients in the low GA-MSCRGPI group had a better prognosis than those in the high group whether they were treated with temozolomide at any time (Fig. 5A). Stratification analysis was further performed according to MGMT promoter status, which has important reference significance for the choice of temozolomide treatment. We obtained a consistent result across all three cohorts: for temozolomide treatment, patients in the low GA-MSCRGPI group had a longer survival time regardless of whether the MGMT promoter was methylated (Fig. 5B, C). Moreover, in patients with radiotherapy, the low GA-MSCRGPI group also had a longer survival time than the high group (Fig. 5D). In the subgroups stratified by different WHO grades, similar results were obtained except for the WHO II subgroups in both the training cohort and validation cohorts (Fig. 5E, and F). Subgroup analysis was not conducted in TCGA-GBM patients due to the limitation of available data. In summary, although GA-MSCRGPI was not an indicator to guide the implementation of chemoradiotherapy, GA-MSCRGPI might be a novel biomarker in predicting chemoradiotherapeutic response in glioma patients.

### Immune characteristics and response to ICI therapy of different GA-MSCRGPI subgroups

**Fig. 6:**
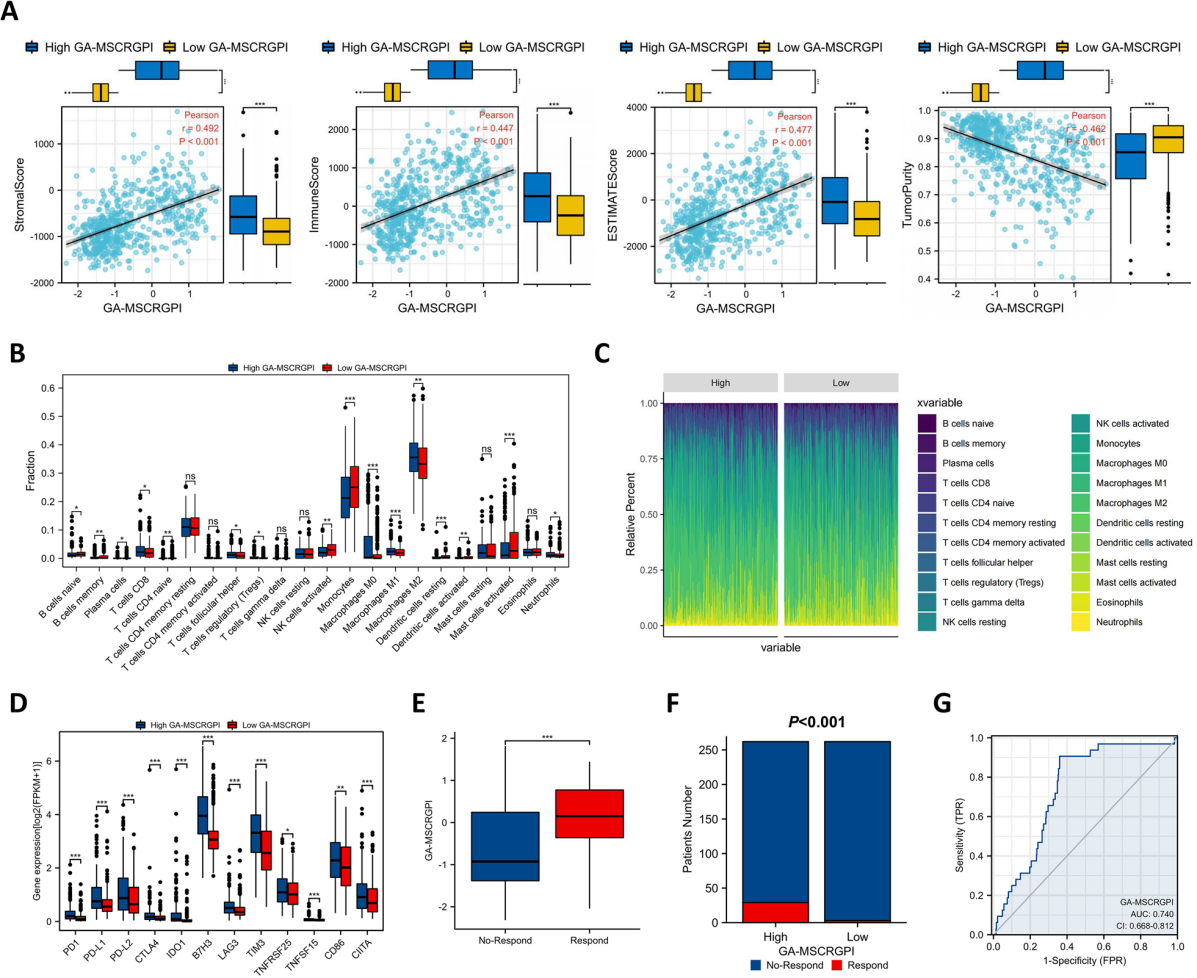
Immune features and response to ICI therapy of different GA-MSCRGPI subgroups. **A** Association between immune score, stromal score, ESTIMATE score, tumor purity, and GA-MSCRGPI and their distribution in the low and high GA-MSCRGPI subgroups. **B** The infiltration of 22 immune cells in the high and low GA-MSCRGPI subgroups. **C** The proportions of 22 immune cells in different GA-MSCRGPI subgroups. **D** The expression of 12 immune checkpoints between different GA-MSCRGPI subgroups. **E** The comparison of GA-MSCRGPI between responders and nonresponders. **F** The distribution of ICI therapy responders in different GA-MSCRGPI subgroups. **G** ROC curve analysis of GA-MSCRGPI in predicting the response to ICI therapy. *p < 0.05, **p < 0.01, ***p < 0.001, and ns No significance

Previous studies have reported that GA-MSCs can secrete a variety of cytokines involved in immune regulation, including IL-6, IL-8, FGF-2 and TGF-β [10, 14]. Moreover, some important immunomodulatory genes, such as CXCL1, CXCL2, CXCL3, CXCL5, CSF3, and CCL20, were also included in the 814 DEGs mentioned above. This indirect evidence suggests that GA-MSCs may be involved in tumor immune regulation. Thus, we preliminarily explored the correlation of prognostic GA-MSCs with the immune landscape of the glioma microenvironment. In the TCGA cohort, the high GA-MSCRGPI group revealed significantly higher immune, stromal and ESTIMATE scores and lower tumor purity than the low group (Fig. 6A). Furthermore, different extents of immune cell infiltration were observed in the high-risk group, with a higher abundance of memory B cells, plasma cells, CD8+ T cells, CD4+ naive T cells, follicular helper T cells, Tregs, M0-type macrophages, M1-type macrophages, M2-type macrophages, activated dendritic cells, and neutrophils but a lower abundance of naive B cells, activated NK cells, monocytes, resting dendritic cells, and activated mast cells (Fig. 6B, C). In addition, we also compared the expression of some representative immune checkpoints (PD-1, PD-L1, PD-L2, CTLA4, IDO1, B7H3, LAG-3, TIM-3, TNFRSF25A, TNFSF15, CD86 and CIIAT) between different GA-MSCRGPI subgroups. As shown above, the high GA-MSCRGPI group had significantly higher expression levels of all twelve immune checkpoints, in which GA-MSCRGPI had a notable relationship with the expression of B7H3 (Fig. 6D). Finally, we used the TIDE algorithm to evaluate the potential response to ICI therapy of different GA-MSCRGPI subgroups. The GA-MSCRGPI of responders was significantly higher than that of nonresponders (Fig. 6E). Moreover, there were more responders to ICI therapy in the high GA-MSCRGPI group than in the low GA-MSCRGPI group (Fig. 6F). The diagnostic ROC curves revealed high accuracy of the GA-MSCRGPI in predicting the response to ICI therapy (Fig. 6G). In general, the GA-MSCRGPI may be an indicator for predicting the response to ICI therapy in glioma.

### Mutation profile of different GA-MSCRGPI subgroups

**Fig. 7:**
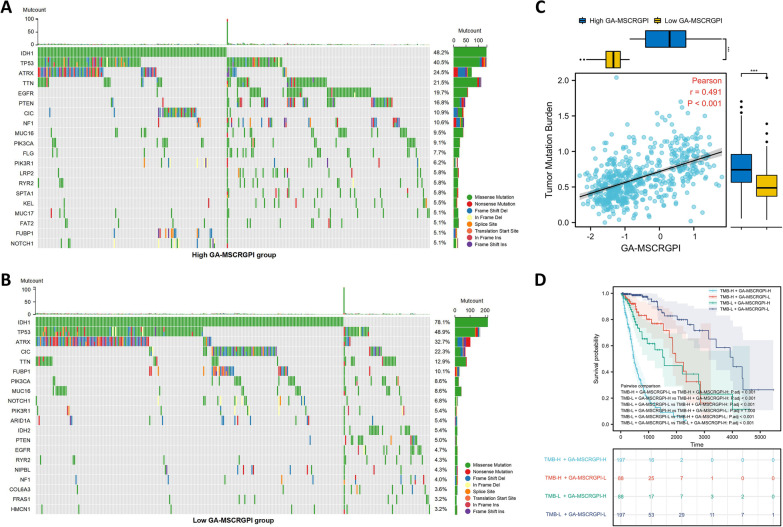
The mutation profile and TMB of different GA-MSCRGPI subgroups. **A, B** Mutation profile in high and low GA-MSCRGPI subgroups. **C** Association between TMB and GA-MSCRGPI and its distribution in the low and high GA-MSCRGPI subgroups. **D** Kaplan‒Meier curves of different TMB and GA-MSCRGPI subgroups for survival. ***p < 0.001

To further investigate GA-MSCRGPI-related mechanisms in glioma, we also analyzed the genetic mutation profile in the TCGA cohort. We identified the top 20 genes with the highest mutation rates in different GA-MSCRGPI subgroups. Although there were no significant differences in overall mutant frequencies, the mutation rate of different mutated genes varied greatly between the low and high GA-MSCRGPI subgroups. The mutation rates of TTN, EGFR, PTEN, and NF1 were higher in the high GA-MSCRGPI subgroup (Fig. 7A), but the mutation rates of IDH1, TP53, ATRX, CIC, and FUBP1 were higher in the low GA-MSCRGPI subgroup (Fig. 7B). In addition, there was a positive correlation between TMB and GA-MSCRGPI, and the TMB of the high GA-MSCRGPI subgroup was significantly higher than that of the low subgroup (Fig. 7C). Finally, the Kaplan–Meier survival curve for the combination of TMB and GA-MSCRGPI showed significant differences in survival outcome, which was the worst for patients with high TMB and high GA-MSCRGPI but the best for patients with low TMB and low GA-MSCRGPI (Fig. 7D).

### Validation of the expression levels of selected GA-MSCRGs

**Fig. 8:**
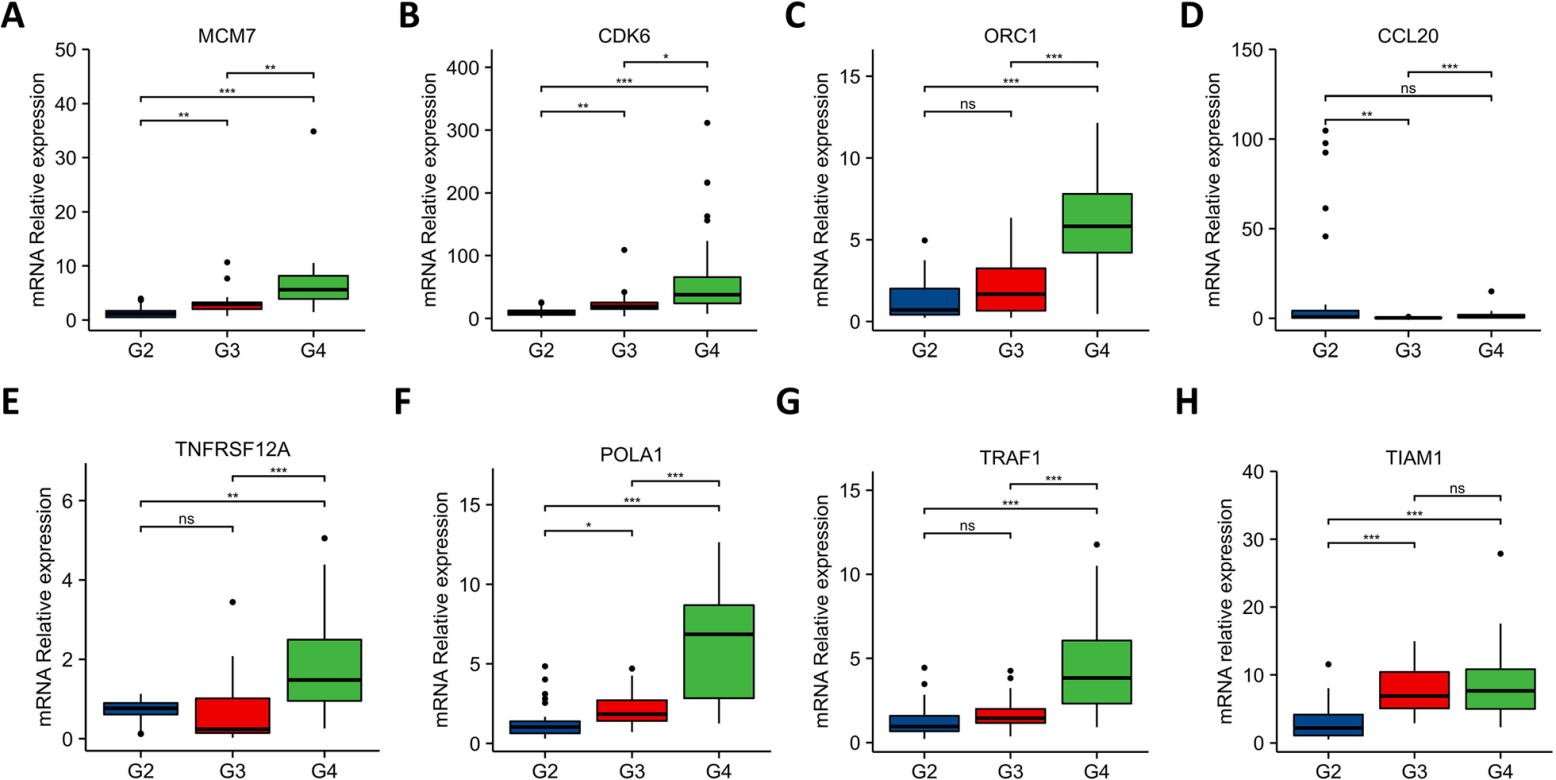
Validation of the expression levels of 8 selected GA-MSCRGs. Expression of MCM7 (**A**), CDK6 (**B**), ORC1 (**C**), CCL20 (**D**), TNFRSF12A (**E**), POLA1 (**F**), TRAF1 (**G**) and TIAM1 (**H**) in our glioma specimen cohorts (G2: WHO grade II, G3: WHO grade III, G4: WHO grade IV, *p < 0.05, **p < 0.01, ***p < 0.001, and ns: no significance)

To further validate the expression patterns of eight selected GA-MSCRGs, we detected their mRNA expression by using qRT‒PCR assay in 78 cases of glioma tissue specimens, which included 26 cases of WHO grade II, 26 cases of WHO grade III and 26 cases of GBM. The expression levels of MCM7, CDK6 and POLA1 showed a remarkably elevated trend according to the grade of the tumor, and differences between all grades were statistically significant (Fig. 8A, B and F). In addition, the expression levels of ORC1, TNFRSF12A, TRAF1 and TIAM1 were also positively correlated with tumor grade to a certain extent (Fig. 8C, E, G and H). In the actual detection process, we found that the expression levels of CCL20 were relatively low in almost all specimens. Their peak periods of the amplification curve were generally later than those of the other indexes. In our results, the expression levels of CCL20 were lowest in grade III glioma, while there was no statistically significant difference between grade II glioma and GBM (Fig. 8D).

## Discussion

In 2015, GA-MSCs were isolated from fresh glioma tissues for the first time[10]. Similar to other types of human mesenchymal stem cells, GA-MSCs have classical cellular morphology, surface markers, and differentiation potential. Meanwhile, GA-MSCs lack expression of CD133 or CD34, which suggests they are not glioma stem cells or endothelial cells. The role of GA-MSCs that exist as stromal cells and enhance the malignant progression of gliomas was confirmed previously [10–12, 15]. In fact, tumor-associated mesenchymal stem cells (TA-MSCs) have been identified in many other types of solid tumors [22]. They play different roles in their respective tumor microenvironments and influence tumor biological behaviors. However, studies using TA-MSCs as prognostic biomarkers have rarely been reported. On the one hand, TA-MSCs are not uniformly distributed in solid tumors [23], so using FCM to analyze the proportion of TA-MSCs in partial tumor masses results in a large error. On the other hand, due to the heterogeneity of MSCs themselves, there are no accurate markers to guide the separation of cell subsets [24]. In the current study, we used the method of GA-MSC isolation combined with intracranial xenograft modeling to obtain GA-MSCRGs and then established a prognostic index for the clinical evaluation of glioma patients. This approach not only avoided the problems mentioned above intelligently, but its efficacy was validated in public databases.

A total of 45 GA-MSCRGs were screened from DEGs obtained in intracranial xenograft tumors by using GSEA combined with univariate Cox regression. Thus, each of these GA-MSCRGs is not only associated with the OS of glioma patients but also reflects the function of GA-MSCs in the glioma microenvironment. Next, eight GA-MSCRGs were further screened to construct the GA-MSCRGPI. Both in the training cohort and validation cohorts, the GA-MSCRGPI showed an excellent capacity in predicting the OS of glioma patients, with worse survival in the GA-MSCRGPI high group and better survival in the GA-MSCRGPI low group. Moreover, the level of GA-MSCRGPI is not only associated with some clinicopathological characteristics, such as age, tumor grade, IDH status, and 1P19q status, and its prognostic value is not affected by these characteristics. In addition, based on univariate and multivariate Cox regression analyses, which demonstrated that the GA-MSCRGPI is an independent prognostic factor for OS, we established a nomogram for more accurate clinical application. All of these results illustrated that the GA-MSCRGPI had stable and reliable power for prognosis prediction, and it could be widely applicable to glioma patients with various clinical features, rather than just high-grade features.

GA-MSCRGPI was composed of eight genes: MCM7, CDK6, ORC1, CCL20, TNFRSF12A, POLA1, TRAF1, and TIAM1. Minichromosome maintenance complex component 7 (MCM7) and cyclin dependent kinase 6 (CDK6) are classical cancer-related genes that are essential for genome replication and regulating the cell cycle [25], respectively [25]. In previous studies, it has been widely verified that high expression of MCM7 or CDK6 is closely related to the malignant proliferation of glioma cells [26, 27]. Origin recognition complex subunit 1 (ORC1) encodes a subunit of the origin recognition complex that is essential for the initiation of DNA replication in eukaryotic cells [28]. In glioma, ORC1 overexpression could promote its malignant progression by activating the ERK/JNK signaling pathway [29]. As the sole cytokines incorporated into the construction of GA-MSCRGPI, C–C motif chemokine ligand 20 (CCL20) encodes a ligand for the C–C chemokine receptor CCR6 involved in immunomodulation of glioma cells [30]. Interestingly, previous studies have also reported that the CCL20-CCR6 signaling axis could mediate the immunomodulatory function of MSCs by inducing the adhesion of Th17 cells to MSCs in vitro [31]. In addition, IL-17- and IFN-γ-transformed TA-MSCs have high expression levels of CCL20 mediated by the activated NF-κB signaling pathway [32]. Therefore, despite the lack of relevant experimental evidence, we speculate that CCL20 may be a critical transmitter of crosstalk between GA-MSCs and their microenvironment. Tumor necrosis factor receptor superfamily member 12A (TNFRSF12A) encodes a receptor of TNFSF12/TWEAK, whose engagement could induce TNF receptor-associated factor (TRAF) binding to the cytoplasmic tail of TNFRSF12A and then activate intracellular signal transduction cascades, such as the NF-κB pathway [33], STAT3/5 pathway, AKT2 pathway [34], etc. TNFRSF12A promotes glioma cell migration, invasion, and resistance to chemotherapeutic agents [35]. There have been few studies on the expression and function of TRAF1 in glioma, so the role of TRAF1 in glioma can only be inferred from its interaction function with TNFRSF12A and its cancer-promoting effect in other types of cancers. DNA polymerase alpha 1 catalytic subunit (POLA1) encodes an essential protein in the initiation of DNA synthesis [36]. T-cell lymphoma invasion and metastasis 1 (TIAM1) encodes a RAC1-specific guanine nucleotide exchange factor (GEF), which mediates the exchange of guanosine diphosphate (GDP) for guanosine triphosphate (GTP) and regulates RAC1 signaling pathways that affect tumor cell growth, survival, migration and actin cytoskeletal formation [37]. Current studies on the role of POLA1 or TIAM1 in glioma cells or mesenchymal stem cells are limited; thus, further studies should focus on these issues. On the whole, these well-chosen GA-MSCRGs are mainly involved in regulating DNA replication and the cell cycle, activating the cytokine signaling pathway, activating the NF-κB signaling pathway and mediating immunomodulation, which are partially consistent with the function of GA-MSCs reported by us or others.

To date, the standard treatment for malignant glioma is still surgical resection combined with concurrent chemoradiotherapy. Temozolomide, a kind of first-line chemotherapeutic drug for glioma, encounters drug resistance in almost all patients [38]. The mechanisms of temozolomide resistance mainly derive from many biological processes, including DNA damage repair, autophagy and glioma stem cells [39]. Recently, we reported that the conditioned medium of CD90^Low^ GA-MSCs could stimulate glioma cells to acquire temozolomide resistance in vitro and vivo, which suggested that GA-MSCs may act as an independent factor contributing to glioma temozolomide resistance [15]. In this study, we systematically evaluated the association between GA-MSCRGPI and the efficacy of temozolomide chemotherapy and radiotherapy. Interestingly, our results cannot determine whether a connection exists between the GA-MSCRGPI-based stratification and the efficacy of temozolomide chemotherapy or radiotherapy, but in all patients undergoing temozolomide chemotherapy or radiotherapy, patients with a high GA-MSCRGPI generally have a worse prognosis. Thus, we hold the opinion that the GA-MSCRGPI could be used to evaluate the prognosis of glioma patients undergoing chemoradiotherapy but cannot act as an indicator of choosing chemoradiotherapy. Put another way, whether patients underwent chemoradiotherapy or not, it does not affect the prognostic value of the GA-MSCRGPI. From the clinical perspective, this conclusion makes up for the shortcomings of previous studies to some extent.

Numerous studies have shown that human mesenchymal stem cells have immunomodulatory properties and immunosuppressive actions [40]. In fact, compared to other types of MSCs isolated from normal tissues, TA-MSCs exhibit stronger immunosuppressive activity [22]. Although the immune characteristics of GA-MSCs have not yet been reported, there is still much indirect evidence, as mentioned above, suggesting that GA-MSCs may be involved in glioma immune regulation. In this study, we found that patients with high GA-MSCRGPI had higher immune scores, higher stromal scores, and higher ESTIMATE scores but lower tumor purity. In addition, infiltrated immune cells were also different between the two GA-MSCRGPI subgroups. Patients with high GA-MSCRGPI tended to have more infiltration of Tregs and M2-type macrophages and fewer activated NK cells, which implied that high GA-MSCRGPI was positively correlated with characteristics of immunosuppression. In addition, the high GA-MSCRGPI group had significantly higher expression of immune checkpoints than the low group. In particular, B7H3, the most dramatically changed immune checkpoint, is a potential target of CAR-T products, which exhibit promising efficacy in the treatment of glioblastoma both in vitro and in vivo [41]. Furthermore, we applied the TIDE algorithm to estimate the relationship between GA-MSCRGPI and response to ICI therapy. The results suggested that a higher GA-MSCRGPI reflected a worse response to ICI therapy, and GA-MSCRGPI may be a promising biomarker for predicting the response to ICI therapy in glioma.

It is well known that genetic mutations are closely associated with the malignant nature of gliomas, but the relationship between genetic mutation and GA-MSC infiltration remains unclear. In this study, we found that the mutation of IDH1 was the most frequent in both the high and low GA-MSCRGPI subgroups. However, the mutation of IDH1 was 48.2% in the high GA-MSCRGPI subgroup and 78.1% in the low GA-MSCRGPI subgroup. In addition, mutation rates of EGFR and PTEN were also more notable in the high GA-MSCRGPI subgroup. These mutational differences implied that patients with high GA-MSCRGPI tend to have a worse prognosis. TMB can indirectly reflect the ability and degree of neoantigen production and predict the immunotherapy efficacy of various cancers. Studies have shown that higher TMB is associated with better OS, and higher TMB is associated with a better response to ICI therapy in most types of cancers [42]. Our results suggest that GA-MSCRGPI was positively correlated with TMB levels, and patients with low GA-MSCRGPI and low TMB had better prognosis than others. These results provide indirect evidence for the prognostic ability of GA-MSCRGPI.

Nonetheless, some limitations should be addressed in our study. First, due to the limitation of experimental conditions, we did not design a more ideal patient-derived tumor xenograft (PDX) model. Therefore, the effect of other tumor stromal components on screened GA-MSCRGs cannot be verified. Second, in this study, we focused on the coding RNAs associated with GA-MSCs, while relevant noncoding RNAs were not discussed. Relevant studies need to be carried out in the future. Third, current studies on GA-MSCs are still at a relatively superficial level, and more precise research methods, such as single-cell sequencing, spatial transcriptome sequencing, or PDX models, can help to optimize GA-MSCRGS screening and GA-MSCRGPI construction.

## Conclusion

In summary, our work fills a research gap in the prognostic analysis of GA-MSCs in glioma. The constructed GA-MSCRGPI showed robust power in predicting the survival outcomes of glioma patients and was correlated with the glioma immune microenvironment. To a certain extent, GA-MSCRGPI could also be used as a biomarker to predict the response to chemoradiotherapy and ICI therapy. We expect that our findings could provide valuable insights for subsequent studies and clinical practice.

## Supplementary Information

**Additional file 1**. **Table S1**: The clinicopathological characteristics of 78 patients in our cohort.

**Additional file 2**. **Table S2**: The clinicopathological characteristics of all included patients in the training and validation cohorts.

**Additional file 3**. **Table S3**: Details of all primer sequences.

**Additional file 4**. A total of 814 DEGs in microarray.

**Additional file 5**. **Figure S1**: Univariate Cox regression analysis of 54 DEGs in the TCGA (**A**) and CGGA325 (**B**) cohorts.

**Additional file 6**. **Figure S2**: Validation of the GA-MSCRGPI in the TCGA and CGGA325 cohorts. (**A, E**) The expression comparison of 8 selected GA-MSCRGs between different grade glioma tissues in TCGA and CGGA325 cohort (G2: WHO grade II, G3: WHO grade III, G4: WHO grade IV; **p < 0.01,***p  < 0.001, and ns No significance). (**B, F**) Kaplan‒Meier curves of GA-MSCRGPI subgroups for survival. (**C, G**) The distribution plots of GA-MSCRGPI, survival status and expression of 8 selected GA-MSCRGs. (**D, H**) ROC curve analysis of GA-MSCRGPI in predicting 2-, 3- and 5-year OS.

**Additional file 7**. **Figure S3**: The correlation between GA-MSCRGPI-based stratification and the efficacy of chemoradiotherapy. (**A**) Kaplan‒Meier curves for patients with or without TMZ chemotherapy in the high GA-MSCRGPI group. (**B**) Kaplan‒Meier curves for patients with or without TMZ chemotherapy in the low GA-MSCRGPI group. (**C**) Kaplan‒Meier curves for patients with or without radiotherapy in the high GA-MSCRGPI group. (**D**) Kaplan‒Meier curves for patients with or without radiotherapy in the low GA-MSCRGPI group.

## Data Availability

Publicly available datasets were analyzed in this study. These data can be found here: TCGA-GBM: (https://portal.gdc.cancer.gov/projects/TCGA-GBM). TCGA-LGG: (https://portal.gdc.cancer.gov/projects/TCGA-LGG). CGGA-693 and CGGA-325: (http://www.cgga.org.cn/download.jsp). Other data generated or analyzed during this study are included in this published article (and its supplementary information files). Clariom D microarray raw data are available in the OMIX repository, accession number: OMIX003259 (https://ngdc.cncb.ac.cn/omix/preview/msjPE6j9).

## Abbreviations

GA-MSCs: Glioma-associated mesenchymal stem cells
GA-MSCRGs: Glioma-associated mesenchymal stem cell-related genes
GA-MSCRGPI: Glioma-associated mesenchymal stem cell-related genes prognostic index
IDH: Isocitrate dehydrogenase
MGMT: O-6-methylguanine-DNA methyltransferase
TME: Tumor microenvironment
CAFs: Cancer-associated fibroblasts
GSCs: Glioma stem cells
GSEA: Gene Set Enrichment Analysis
ICI: Immune checkpoint inhibition
RT‒PCR: Quantitative Real-Time polymerase chain reactionq
PBS: Phosphate-buffered saline
CGGA: Chinese Glioma Genome Atlas
TCGA: The Cancer Genome Atlas
OS: Overall survival
FPKM: Fragments Per Kilobase of exon model per Million mapped fragments
Lasso: Least absolute shrinkage and selection operator
DEGs: Differentially expressed genes
KEGG: Kyoto Encyclopedia of Genes and Genomes
ROC: Receiver operating characteristic
C-index: The concordance index
AUC: Area Under Curve
TMZ: Temozolomide
WHO: World Health Organization
TA-MSCs: Tumor-associated mesenchymal stem cells
MCM7: Minichromosome maintenance complex component 7
CDK6: Cyclin-dependent kinase 6
ORC1: Origin recognition complex subunit 1
CCL20: C–C motif chemokine ligand 20
TNFRSF12A: Tumor necrosis factor receptor superfamily member 12A
TRAF1: TNF receptor-associated factors 1
POLA1: DNA polymerase alpha 1 catalytic subunit
TIAM1: T-cell lymphoma invasion and metastasis 1
GEF: Guanine nucleotide exchange factor
GDP: Guanosine diphosphate
GTP: Guanosine triphosphate
TIDE: Tumor Immune Dysfunction and Exclusion
PDX: Patient-derived tumor xenograft
H&E: Hematoxylin–eosin staining
HR: Hazard Ratio
CI: Confidence interval
