# Supplementary figures and images for: Development and validation of a glioma-associated mesenchymal stem cell-related gene prognostic index for predicting prognosis and guiding individualized therapy in glioma
Source: Stem Cell Res Ther. 2023 Apr 1;14:56. doi: 10.1186/s13287-023-03285-9 (PMC10068170; doi:10.1186/s13287-023-03285-9)

A

## Univariate Cox analysis in TCGA

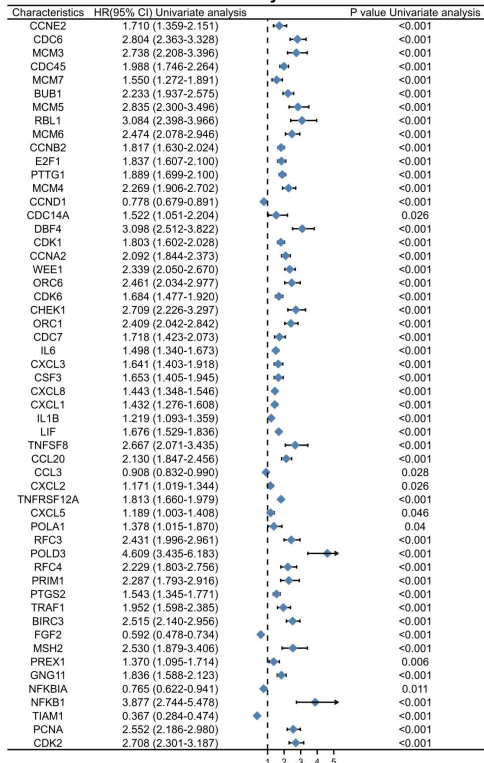

B

## Univariate Cox analysis in CGGA325

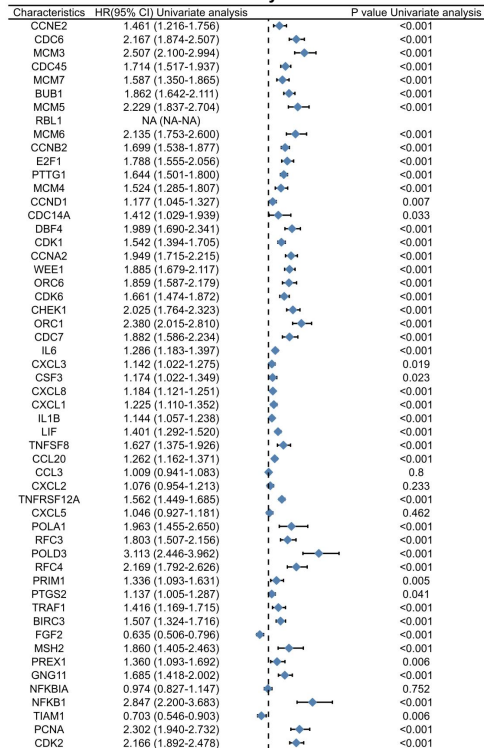

Supplement: Supplementary file 5 — Additional file 5. Figure S1: Univariate Cox regression analysis of 54 DEGs in the TCGA (A) and CGGA325 (B) cohorts. [file 13287_2023_3285_MOESM5_ESM.pdf]

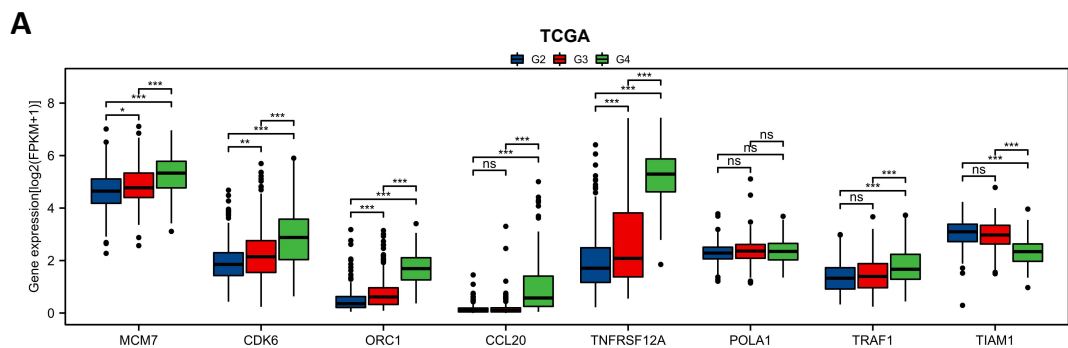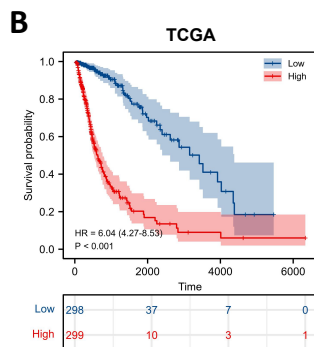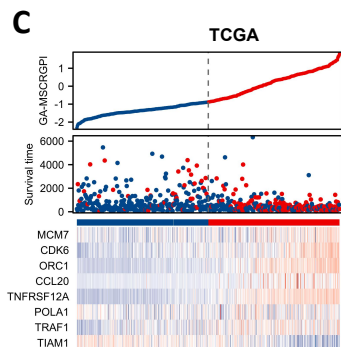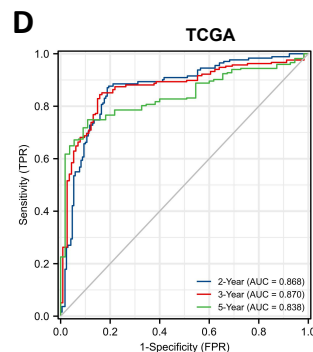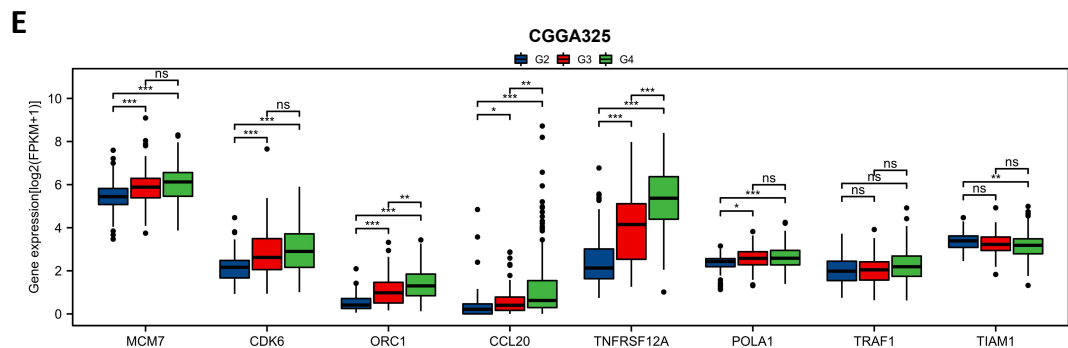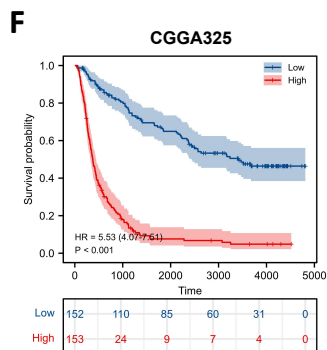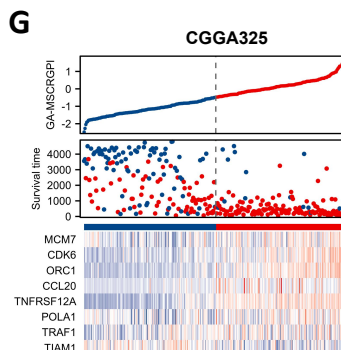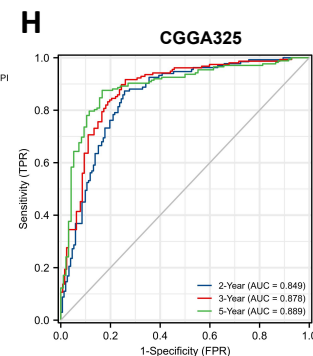

Supplement: Supplementary file 6 — Additional file 6. Figure S2: Validation of the GA-MSCRGPI in the TCGA and CGGA325 cohorts. (A, E) The expression comparison of 8 selected GA-MSCRGs between different grade glioma tissues in TCGA and CGGA325 cohort (G2: WHO grade II, G3: WHO grade III, G4: WHO grade IV; **p < 0.01,***p < 0.001, and ns No significance). (B, F) Kaplan‒Meier curves of GA-MSCRGPI subgroups for survival. (C, G) The distribution plots of GA-MSCRGPI, survival status and expression of 8 selected GA-MSCRGs. (D, H) ROC curve analysis of GA-MSCRGPI in predicting 2-, 3- and 5-year OS. [file 13287_2023_3285_MOESM6_ESM.pdf]

**A****High GA-MSCRGPI Group**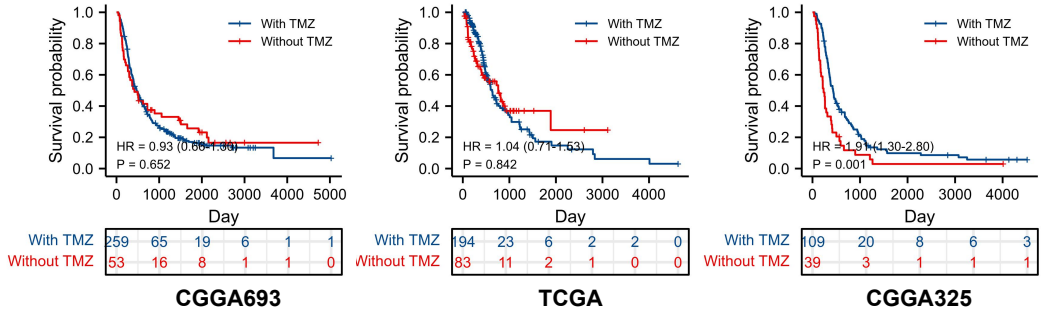**B****Low GA-MSCRGPI Group**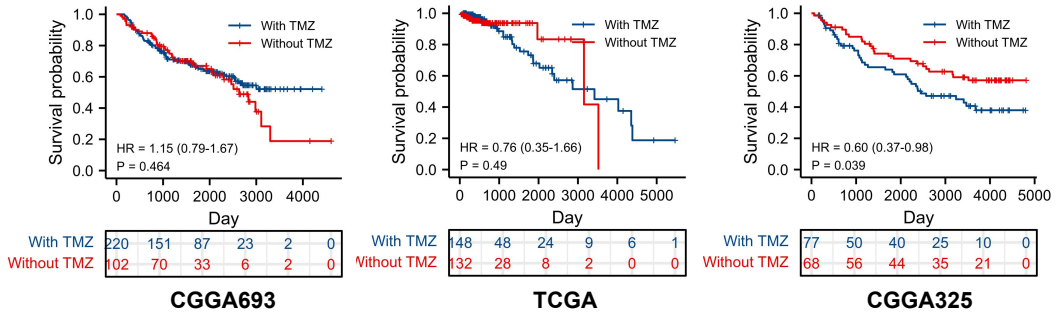**C****High GA-MSCRGPI Group**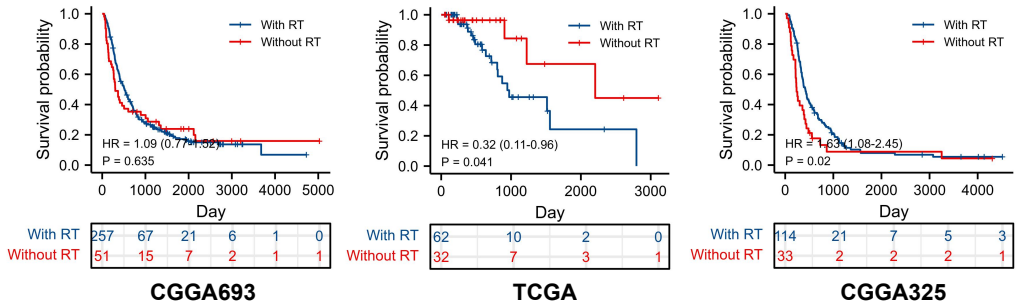**D****Low GA-MSCRGPI Group**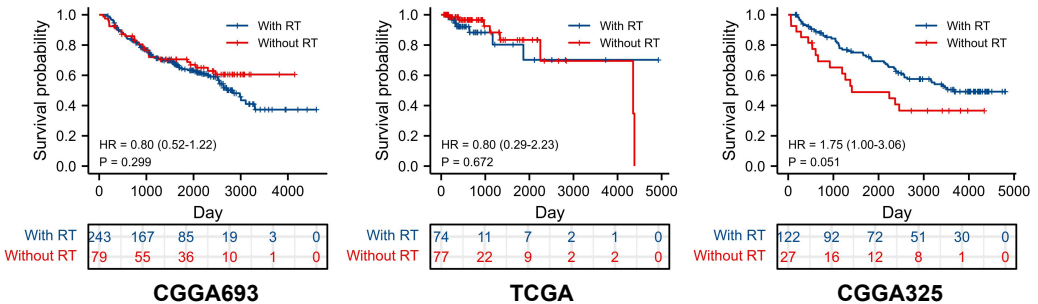

Supplement: Supplementary file 7 — Additional file 7. Figure S3: The correlation between GA-MSCRGPI-based stratification and the efficacy of chemoradiotherapy. (A) Kaplan‒Meier curves for patients with or without TMZ chemotherapy in the high GA-MSCRGPI group. (B) Kaplan‒Meier curves for patients with or without TMZ chemotherapy in the low GA-MSCRGPI group. (C) Kaplan‒Meier curves for patients with or without radiotherapy in the high GA-MSCRGPI group. (D) Kaplan‒Meier curves for patients with or without radiotherapy in the low GA-MSCRGPI group. [file 13287_2023_3285_MOESM7_ESM.pdf]
